# Speech analysis for acute decompensated heart failure: reporting a pilot study in Germany

**DOI:** 10.3389/fdgth.2026.1885715

**Published:** 2026-08-12

**Authors:** Andreas Triantafyllopoulos, Wolfgang Mayr, Anton Batliner, Lana Lempkens, Björn W. Schuller, Thomas M. Berghaus

**Affiliations:** 1 Chair of Health Informatics (CHI), University Hospital of the Technical University of Munich, Munich, Germany; 2 Munich Center for Machine Learning (MCML), Munich, Germany; 3 Department of Cardiology, Respiratory Medicine and Intensive Care, University Hospital Augsburg, Augsburg, Germany; 4 Group on Language, Audio, & Music (GLAM), Imperial College London, London, United Kingdom; 5 Medical Faculty, Ludwig Maximilians University of Munich, Munich, Germany

**Keywords:** digital health, feature interpretation, heart failure, pathological speech, personalization

## Abstract

Acute decompensated heart failure (ADHF) has been shown to affect breathing and speech production. We present a new German speech dataset and conduct a pilot study to show the feasibility of using speech to distinguish between decompensated and recompensated ADHF. We achieve accuracy rates of 77% for this binary classification using read speech, with the use of segmented phrases scoring at 75% and sustained vowels trailing far behind at 52%. Our feature analysis shows a reduction in breathiness and an increase in articulation stability during recompensation. Overall, our results show the promise of speech analysis in the monitoring of ADHF and demonstrate that further efforts should be invested to obtain large-scale data and replicate our results in more heterogeneous populations.

## 1 Introduction

Acute decompensated heart failure (ADHF) is a leading cause of unplanned hospital admissions in older adults and imposes substantial economic burden on healthcare systems (1). Pathophysiologically, heart failure occurs when the heart fails to supply sufficient blood and oxygen to the body (2). Globally, over 37 million individuals are affected by heart failure (3). Clinically, heart failure manifests through symptoms such as dyspnea and edema in systemic and/or pulmonary tissues. ADHF patients face elevated in-hospital mortality, frequent readmissions, and generally poor clinical trajectories, significantly impairing their physical and social functioning and reducing quality of life (4).

**Figure 1:**
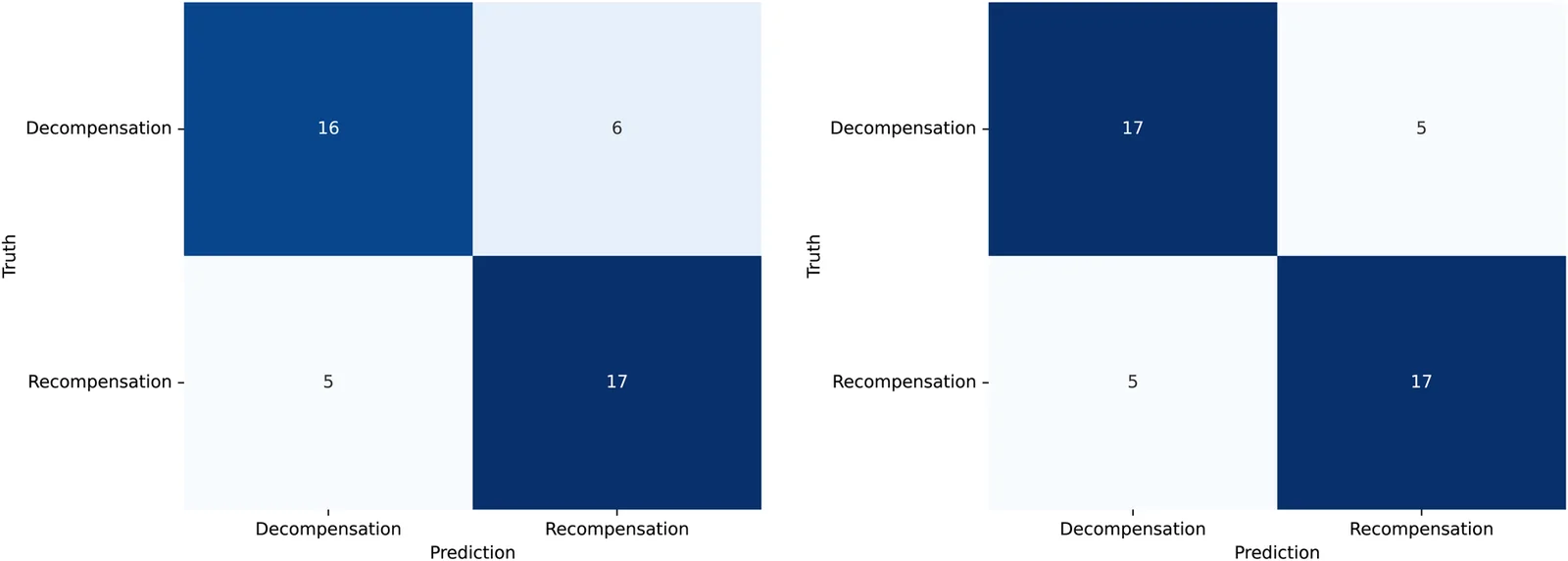
Confusion matrix for phrase-level (left) and story-level (right) eGeMAPS features, aggregated over all folds.

Although various biomarkers are used to monitor disease progression, current tools for early detection of decompensation remain limited. For example, daily weight monitoring is a simple and non-invasive method for patients to detect fluid retention at home (1). However, weight gain typically occurs late in the decompensation process and may be absent or overlooked. In fact, a previous study found that only around 30% of ADHF patients experienced weight gain in the week prior to hospitalization, and only about 50% noticed worsening of dyspnea or edema (5). This highlights the need for more sensitive, non-invasive methods capable of detecting early signs of decompensation (6).

In recent years, voice analysis has emerged as a promising tool in this context. Since phonation depends on pulmonary airflow, vocal cord vibration, and neuromuscular control, voice can reflect changes in respiratory and cardiovascular function. Fluid accumulation in the vocal folds or lungs, as well as altered heart rate or blood pressure, can affect speech production—resulting in changes in pitch, voice stability, speech tempo, and pause patterns (7, 8). Even small amounts of laryngeal edema may lead to detectable shifts in voice quality (8). Several studies have already explored this potential (7–13). For instance, Murton et al. (8) identified changes in vocal characteristics such as increased speech irregularity and reduced fundamental frequency during acute decompensated episodes, which improved after diuretic treatment. Similarly, Schöbi et al. (10) analyzed 68 patients with ADHF and 36 with stable heart failure and found significantly prolonged pause durations and slower speech in the ADHF group. Notably, the pause ratio correlated positively with NT-proBNP levels, suggesting a link between voice changes and cardiac status independent of overt symptoms (10). Another approach, using the HearO app, was able to distinguish “wet” (decompensated) from “dry” (compensated) states in heart failure patients with high accuracy, demonstrating changes of up to 218% in certain speech parameters (14).

This work presents a pilot study with a new dataset of German speech collected at the University Hospital of Augsburg/Germany. We reproduce a recent speech analysis pipeline that we proposed for the detection of exacerbation vs recovery in chronic obstructive pulmonary disease (COPD) patients. Our primary goal is to investigate the feasibility of using the same pipeline to distinguish between decompensation and recompensation in ADHF, while additionally interpreting the key acoustic features of our analysis.

## 2 Methodology

### 2.1 Data collection

In this single centre study, non-consecutive patients with ADHF were prospectively enrolled at the University Hospital of Augsburg within 48 h after emergency admission between September 2024 and May 2025. The local ethics committee approved the study on July 18, 2024 (Project-Nr.: 24-0490). Only patients older than 18 years, with a diagnosed ADHF, able to sit and read a short text without any need of ventilation or oxygen supply during the time of recording, were included. Exclusion criteria were relevant decompensations or exacerbations of respiratory diseases, ear, nose and throat (ENT) comorbidities, active cancer disease or any palliative situation. Clinical baseline data were extracted from medical records and through standardized patient interviews. Two recordings—one during and the second after an acute exacerbation—took place bedside using a smartphone equipped with a custom Android app developed by the authors and a RØDE smartLav+ lapel microphone, attached to the clothes of the patient, at chest level. Data were stored in raw format with a 16-bit signed integer representation and a sampling rate of 44,100 Hz. They were subsequently converted to a WAV representation using *sox*. We used a single Samsung Galaxy A25 phone to conduct all recordings. The recordings were supervised by the 2nd and 4th authors and were conducted in the patients’ rooms, with as little background noise as possible.

All patients obtained their individual home medication and a standard treatment of their ADHF, mainly intravenous diuretics. Some patients needed non-invasive ventilation, which was paused for the time of recording, as well as oxygen supply. The two recordings were performed under controlled settings in a quiet surrounding without any background noises. Patients were required to produce: (a) a set of sustained vowels (/a:/, /e:/, /i:/, /o:/, /u:/); (b) a few spontaneous utterances; (c) (forced) coughing; (d) normal breathing; (e) reading a standardized text “Der Nordwind und die Sonne” (The Northwind and the Sun, NuS).

### 2.2 Clinical assessment of disease severity

Echocardiography was performed once for each patient under stable clinical conditions during the hospital stay. Left ventricular ejection fraction, diastolic function, as well as the type and severity of valvular defects were recorded. The modified Medical Research Council (mMRC) scale was used to evaluate dyspnea at admission and at discharge from the hospital stay. The mMRC is a validated tool that measures the degree of breathlessness during daily activities on a scale from 0 (only breathless with strenuous exercise) to 4 (too breathless to leave the house or breathless when dressing) (15). Health status was assessed using the 23-item Kansas City Cardiomyopathy Questionnaire (KCCQ-23), a validated, disease-specific instrument measuring physical limitation, symptom burden, social function, and quality of life in patients with heart failure. Scores range from 0 to 100, with higher scores indicating a better health status (16). At discharge, the questionnaire was administered again, but only selected items (questions 1, 2, 8, 13, and 15) were used to specifically capture changes in key domains. In addition, laboratory values such as NT-proBNP, hs-Troponin, and creatinine were taken from the patient’s medical record at the time of admission. An additional blood sample as part of the study was not necessary. Finally, clinical data, such as current smoking status, pack-years, existing peripheral edema or pulmonary congestion or effusion, triggers for the current decompensation, underlying cardiac diseases, other comorbidities and the duration of the current hospitalization, were extracted from the patient’s medical record or through standardized interviews.

Recompensation from ADHF was evaluated clinically and was defined by the following criteria: absence or best possible relief of symptoms like dyspnea, lower limb swelling, elevated jugular venous pressure or pulmonary congestion. In addition, patients should have regained their body weight before ADHF.

### 2.3 Speech processing

The main goal of this study is to classify the speech of patients in two states of the disease: during ADHF and during recompensation. This was done by training an ML model using the speech features we extracted from the three units of analysis: (a) sustained vowels, (b) phrases, (c) story. We evaluated our performance using balanced accuracy, F1-score and the Receiver Operating Characteristic Area Under the Curve (ROC-AUC). Our dataset had a perfect class balance (matched number of exacerbations and recompensations) so the weighted versions of those metrics are equal to the unweighted versions.

**Preprocessing:** Speech recordings were obtained for each of the different voice tests (sustained vowels, breathing, coughing, read speech). For the purposes of this study, we only focused on sustained vowels and read speech. Read speech was subsequently segmented with the web tool MAUS (Munich AUtomatic Segmentation) (17, 18) using forced alignment and individual words manually assigned to the prosodic phrases corresponding to breaks in the target text.

**Units of analyses**: We used the entire **story** (19), i.e., without segmentation, as well as the set of segmented **phrases** (20). We also used all sustained **vowels** (21). In the case of phrases and vowels, we computed a max vote for each speaker in each phase.

**Speech features:** We computed speech features for each audio using the open-source Speech and Music Interpretation by Large-space Extraction toolkit (openSMILE) (22). We extracted the extended Geneva Minimalistic Acoustic Descriptor Set (eGeMAPS) (23)—a small set of 88 acoustic parameters that has previously been shown to contain relevant information for respiratory diseases (19).

**Rhythm features:** We constructed a small, interpretable feature set comprising global speech rate features, local and global duration variability features, and surface phonological features:

- **Speech rate features:** Tempo features include the ***T**otal duration* (T), the ***T**otal **s**peech duration* (T${}_{s}$; as detected by MAUS), the *rate of **Ph**onemes over **T**otal duration* (Ph/T), and the *rate of **Ph**onemes over **T**otal **s**peech duration* (Ph/T${}_{s}$).
- **Local variability features:** These are the ***r**aw* and ***n**ormalised* ***P**airwise* ***V**ariability* ***I**ndices* computed on subsequent ***v**ocalic* and ***c**onsonantal* segments: *rPVIv*, *nPVIv*, *rPVIc*, and *nPVIc*. These indices compute the average durational difference between successive vocalic/consonantal segments, with the normalisation intended to reduce the impact of speaking rate. See Grabe and Low (24) for details on implementation.
- **Global variability features:** One prominent set of global variability measures was introduced by Ramus et al. (25) and includes the *total **V**owel duration over total duration* (%V) and the *standard deviation of **V**ocalic/**C**onsonantal segment durations* (δV/δC). We redundantly include *total **C**onsonant duration over total duration* (%C) and *total **P**ause duration over total duration* (%P=1−%V−%C) as in our pathological speech data, we expect pauses to play a decisive role. Similar to nPVI, a normalisation over total duration (i.e., the **C**oefficient of **V**ariation; CV(V)/CV(C)) was later introduced to reduce the influence of speech rate (26, 27).

**Standardization:** As speaker standardization was found to substantially improve performance in previous work (20), we used it in our study as well. It entails the computation of standardization parameters (mean and standard deviation) separately for each speaker. Note that this computes separate standardization parameters for training and testing folds, with a distinct standardization applied to each fold. To compute standardization parameters for each speaker, we use all available read speech or sustained vowel data from them.

**Feature Analysis:** We additionally conducted an exploratory feature analysis on the most importance acoustic features. Our decision process for this exploratory analysis was as follows: Based on our classification results (see below) we identified read speech as the most informative data stream and eGeMAPS as the most predictive features. We thus focused on those features for brevity. When eGeMAPS features are extracted from the entire story, which was our best classification configuration, they implicitly capture durational variability and are heavily influenced by pauses. Given that the goal of our exploratory feature analysis is to identify interpretable features, we decided to focus on the phrase-based segmentation, which was found to give marginally worse classification performance. Our segmentation resulted in 20 phrases per file, with one feature vector per phrase. These feature vectors were averaged to obtain one feature vector per file to avoid repeated testing.

We then computed Cohen’s d as a measure of effect size. We also conducted a Wilcoxon signed-rank test and used Bonferroni-Hochberg adjustment to correct for multiple comparisons. The results of this hypothesis testing are reported purely for reasons of transparency. However, we strongly advocate against the use of hypothesis testing for interpreting results, in line with the statement of the American Statistical Association on the inherent and unavoidable limitations of null hypothesis testing (28). Note, for instance, that while we only report the results of a Wilcoxon test for this constellation (averaged phrase-level eGeMAPS features), we also conducted tests for sustained vowels (each vowel separately), story-level eGeMAPS features, and non-averaged phrase-level eGeMAPS features. As our study was intentionally exploratory, we did not shy away from Gelman’s “garden of forking paths” (29) and made pragmatic choices at every step of the process. Future research could try to replicate our findings in a confirmatory setup.

**Modelling:** We employed a nested leave-one-speaker-out cross-validation, whereby data from every speaker is used exactly once for testing, each time using the data of all other speakers for training. This process was used to train a support vector machine (SVM) classifier, where we optimized the cost parameter (.0001, .0005, .001, .005, .01, .05, .1, .5, 1) and kernel function (linear, polynomial, radial basis function (RBF)) in a grid search manner. These parameters were always optimized on the development partition, which was created by splitting the training speakers into two speaker-disjoint sets. This inner validation loop was conducted by splitting the development partition into two folds and cycling through them for training/validation. Once the optimal set of parameters was identified (based on development set performance), we trained a final model on the entire training data for each fold. This process was repeated independently for each unit of analysis (sustained vowels, phrases, story) and feature set (eGeMAPS, rhythm).

## 3 Results

### 3.1 Cohort statistics

Patient metadata are shown in Table 1. We recruited a total of 22 patients who got admitted with an ADHF to the University Hospital of Augsburg. All of the participants completed two recordings, one within 48 h after admission to the hospital and one 24 h before discharge. 40.9% of our patients were male, 13.6% of our patients were current smokers, with an average of 22 pack-years. The average hospital stay was 10.1 days. The average weight loss through diuretic therapy during the hospital stay was 3.6 kg. 77% of the participants had peripheral edema, and 31% had pulmonary venous congestion or pleural effusions. Clinical variables are shown in Table 1 while self-reported questionnaires are reported in Table 2. The average Left Ventricular Ejection Fraction (LVEF) was 36.8% and the mean NT-proBNP at admission was 11,502 pg/mL. 32% of the patients had valvular heart disease as the underlying cardiac condition, 50% had Heart Failure with reduced Ejection Fraction (HFrEF), 4.5% had Heart Failure with mildly reduced Ejection Fraction (HFmrEF), 4.5% had Heart Failure with preserved Ejection Fraction (HFpEF), and 2% had pulmonary hypertension. The cause of decompensation was attributed to valvular heart disease in 59% of cases, hypertension in 18%, arrhythmias in 14%, and infections in 2% of the cases. The average NYHA class was 3.4 at the time of admission and 2.8 at discharge. The overall KCCQ score at admission was on average 54.6, with the selected questions scoring an average of 27.1. At discharge, the selected questions KCCQ score improved to 39.5.

**Table 1:** Clinical metadata recorded for patients.

Variable	N	Mean (std.)
Age [years]	22	70.6 (12.2)
Pack-years	22	18.3 (22.2)
Weight [kg]	22	81.8 (22.2)
Height [cm]	22	167.2 (7.0)
Hospitalisation [days]	22	10.1 (5.1)
Weight loss during stay [kg]	22	3.6 (2.6)
Systolic RR [mmHg]	22	123.9 (26.9)
Diastolic RR [mmHg]	22	71.3 (16.7)
Heartrate [BPM]	22	90.5 (20.7)
spO2 [%]	22	93.6 (3.4)
LVEF${}^{*}$ [%]	22	36.8 (12.3)
proBNP [pg/ml]	22	11,502 (8,573)
hsTroponin [pg/ml]	12	104.6 (154.7)
Creatinine [mg/dl]	22	1.6 (0.8)

LVEF and weight loss measured during hospital stay, all other values at admission. Values reported as mean and standard deviation (std.).

**Table 2:** Self-reported questionnaires administered shortly after admission and shortly before discharge.

Variable	Admission	Discharge
NYHA	3.4 (0.8)	2.8 (0.7)
KCCQ	27.1 (12.1)	39.5 (12.3)

### 3.2 Speech analysis

Classification results are shown in Table 3 and confusion matrices in Figure 1. The best-performing speech models rely on eGeMAPS extracted on the entire story, with an accuracy of 77%, closely followed by eGeMAPS extracted on the individual phrases with 75%. Rhythm features score substantially below, with an accuracy of 64%, whereas the use of sustained vowels instead of read speech results in an accuracy of only 52%, thus only marginally over chance-level performance (50%).

**Table 3:** SVM classification result on different units of analysis using leave-one-speaker-out cross-validation.

Features	Speech units	Accuracy [%]	F1-score [%]	ROC-AUC [%]
*eGeMAPS*	Sustained Vowels	52 [36-68]	52 [36-67]	64 [47-81]
*eGeMAPS*	Phrases	75 [62-88]	75 [61-86]	73 [55-90]
*eGeMAPS*	Story	77 [63-89]	77 [63-89]	74 [56-89]
*Rhythm*	Story	64 [49-77]	64 [48-77]	58 [41-75]

Results are collected over all folds and presented with 95% bootstrap confidence intervals. We present accuracy, F1-score and the Receiver Operating Characteristic Area Under the Curve (ROC-AUC). Note that classes are perfectly balanced so accuracy is equivalent to balanced accuracy. F1-score corresponds to the harmonic mean between precision and recall.

**Figure 2:**
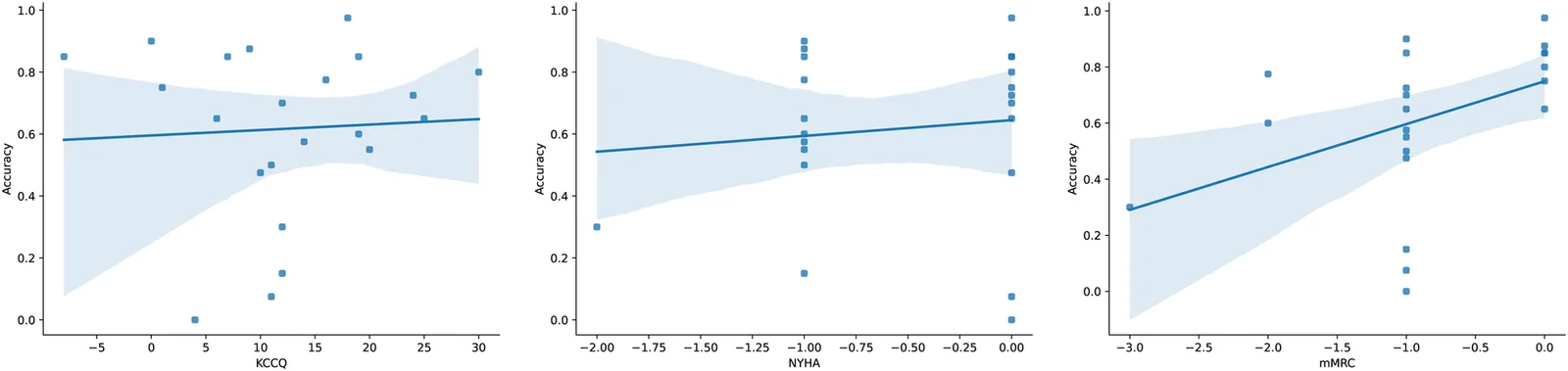
Speaker-level performance (accuracy) vs. clinical metadata. Each dot represents the balanced accuracy of each speaker vs. their change in each clinical variable (KCCQ, NYHA, mMRC) between discharge and exacerbation. Pearson correlation with KCCQ (ρ=.06) and NYHA (ρ=.11) is negligible, while being moderate for mMRC (ρ=.43).

We additionally test the performance per speaker compared to the observed change in self-reported scales (KCCQ, NYHA, mMRC). For this, we compute the accuracy per speaker on the phrase-level data, where we have 40 samples per speaker. Results are shown in Figure 2, where we observe that speaker-level performance can vary between an accuracy of 0% and 100%, indicating that our model can fail catastrophically for some speakers over others. Accuracy shows a moderate correlation with change in mMRC (ρ=.43), which agrees with our intuition that a change in perceived dyspnea will also be detectable in speech. In contrast, more lifestyle-oriented questionnaires, like KCCQ or NYHA, show negligible correlations with speaker-level performance.

Table 4 presents effect sizes (Cohen’s d) for acoustic features in the recompensated vs. the decompensated state. We only show and interpret values that are >.4, i.e., moderate effect sizes. No *p*-values were below the .05 significance level after a Wilcoxon-rank test and Benjamini-Hochberg correction.

**Table 4:** Cohen’s d when comparing recompensated vs. decompensated speech.

Feature	Cohen’s d
$\sigma (MFCC4_{V})$	−.54
μ(MFCC3)	−.50
$\sigma (F1_{f})$	−.49
$\mu (AR_{UV})$	−.45
$\mu (MFCC3_{V})$	−.44
$\sigma (F1_{b})$	−.43
$\mu (MFCC2_{V})$	.41
$\mu (HI_{V})$	.43
μ(MFCC2)	.47
μ(H1−A3)	.58

Only showing features with at least a moderate effect d>.4. Features are the 2nd (MFCC2), 3rd (MFCC3), 4th (MFCC4) Mel-frequency cepstral coefficients, the frequency and bandwidth of the first formant ($F1_{f}/F1_{b}$), the alpha ratio (AR), the Hammarberg index (HI) and the difference between the 1st harmonic and 3rd formant (H1−A3). The use of V or UV specifies that the feature was computed only within voiced or unvoiced segments, respectively. μ(⋅) denotes the mean and σ(⋅) the standard deviation.

During recompensation, we observe a decrease in the standard deviation of the third and fourth MFCCs, μ(MFCC3), $\mu (MFCC3_{V})$, and μ(MFCC4), and a decrease in the variability of the first formant frequency, $\sigma (F1_{f})$, and the variability of its bandwidth, $\sigma (F1_{b})$, all indicative of higher articulatory stability during speaking. In addition, we observe an energy shift from higher towards lower frequencies, evident in an increase of the Hammarberg Index in voiced segments, $\mu (HI_{V})$, which captures the ratio of higher to lower frequencies, shown also in the decrease of the alpha ratio in unvoiced segments, $\mu (AR_{UV})$,^1^ and in the increase of the second MFCC, both overall, μ(MFCC2), and in voiced segments, $\mu (MFCC2_{v})$. These changes in the distribution of frequencies are indicative of a reduction in breathiness, as breathiness manifests as high-frequency content. Finally, there is an increase in the energy of the first harmonic over the third formant, which can also be interpreted as a reduction in creakiness.

## 4 Discussion

Our results indicate that speech can be successfully used to distinguish between decompensation and recompensation in ADHF patients. Moreover, demonstrating the impact of ADHF on speech paves the way for investigating it as a *prognostic* biomarker – i.e., for the detection of decompensation before it occurs (12).

Overall, our recognition results are lower than those reported in previous work (13); however, Bäuser et al. (13) focused on the classification of ADHF patients vs. healthy controls, which is a different—and, presumably, easier—problem than classification of disease stages. This illustrates that the more granular classification of pathology remains a more challenging task than a simple categorization between healthy vs. pathological. In terms of features, we did not observe an impact on F0, which was pervasive in previous work (13). We did, however, find a reduction in creakiness, similar to Murton et al. (8). Our work was submitted concurrently with Riehle et al. (30), who found a combination of sustained vowels and breathing features (related to pause length) to yield the best performance.

More broadly, our results echo our previous work on the classification of decompensation vs. recompensation in COPD (19, 21). There, as here, we found that sustained vowels underperform phrases (21) and that using the entire story results in higher recognition rates (19). Crucially, we observe a similar impact of COPD and ADHF decompensations on patient speech, manifesting as an increase in breathiness and reduced articulatory control (20). Future work should thus focus on elucidating whether the impact of each disease has unique characteristics, or whether we are implicitly distingushing between different degrees of disease severity, without distinguishing between different diagnostic categories. This investigation would have important ramifications for the future of speech analysis in clinical research, given the necessity to tell apart different disorders.

### 4.1 Limitations

A major limitation of our work is the small sample size. This reflects the challenges of conducting research in naturalistic setups. Nevertheless, similar sample sizes have been used to demonstrate feasibility in prior work (20). We consider this a limitation that can be improved by resuming the data collection in a subsequent study. Given the recent interest in ADHF detection using speech, future studies could attempt a confirmatory replication of our (and other) exploratory findings in a multi-site setup in the presence of comorbidities, thus reflecting real-world scenarios for such a technology.

## 5 Conclusion

We presented a pilot study on classifying decompensated vs recompensated speech of German patients. Our machine learning experiments yielded a top accuracy of 77%, showcasing the feasibility of the approach. Our accompanying feature analysis revealed that the highest impact is seen on breathiness (energy in high frequencies) and articulatory control (formant dispersion). Future work should verify these findings in larger, multi-site cohorts and investigate the utility of speech biomarkers as prognostic factors of decompensation.

## Data Availability

The datasets presented in this article are not readily available because data cannot be made available due to data protection regulations. Anonymized features and labels can be made available upon reasonable request to the corresponding author. Requests to access the datasets should be directed to andreas.triantafyllopoulos@tum.de.

## Footnotes

^1^Eyben et al. (23) present the alpha ratio as the ratio of the sum of energies between [0 Hz–1 kHz] and [1 kHz–5 kHz], however, the *openSMILE* code computes the inverse of this ratio (22).
